# Development of Immunochromatographic Strip for Detection of αB-VxXXIVA-Conotoxin Based on 5E4 Monoclonal Antibody

**DOI:** 10.3390/toxins14030191

**Published:** 2022-03-04

**Authors:** Hengkun Tang, Haimei Liu, Rui Chen, Yehong Gao, Mingke Dong, Sumei Ling, Rongzhi Wang, Shihua Wang

**Affiliations:** Key Laboratory of Pathogenic Fungi and Mycotoxins of Fujian Province, Key Laboratory of Biopesticide and Chemical Biology of Education Ministry, School of Life Sciences, Fujian Agriculture and Forestry University, Fuzhou 350002, China; tanghengkun@fafu.edu.cn (H.T.); wuhuijuan@fafu.edu.cn (H.L.); chenrui@fafu.edu.cn (R.C.); gaoyehong@fafu.edu.cn (Y.G.); dmkhubei@fafu.edu.cn (M.D.); lsmpu2008@m.fafu.edu.cn (S.L.)

**Keywords:** αB-VxXXIVA-conotoxin, monoclonal antibody, AuNPs-based strip, AuNFs-based strip, detection

## Abstract

Given the application of αB-VxXXIVA-conotoxin (αB-CTX) in analgesics and cancer chemotherapeutics, and its threat to humans, it is urgent to develop a rapid, effective and accurate method for the analysis and detection of αB-CTX in real shellfish and medicine drug samples. In the present study, two different immunochromatographic strips were established for αB-CTX detection, based on the monoclonal antibody 5E4 against αB-CTX, and the visual limits of detection (vLOD) for the colloidal gold nanoparticles-based strip (AuNPs-based strip) and nanoflowers-based strip (AuNFs-based strip) were 4 μg/mL and 1.5 μg/mL, respectively. The developed AuNPs-/AuNFs-based strips have good specificity and accuracy, and the detection results were analyzed in less than 10 min, without using an instrument. In view of the excellent repeatability and usability, the established methods could be applied to detect and analyze the content of αB-CTX in real samples.

## 1. Introduction

Conotoxins are the peptidic components of the venom that is mainly secreted by marine cone snails. As a kind of neurotoxin, these peptides display unprecedented potency and selectivity for their molecular targets, including ion channels, membrane receptors and neurotransmitter transporters [1]. These significant features have made conotoxins as excellent pharmacological probes, drug leads and therapeutics [2,3]. cDNA sequencing is now the primary method for the identification of new conotoxins [4], and conotoxins are further classified into different pharmacological categories, based on the interaction of receptors/targets with conotoxins, such as sodium channel-targeted conotoxins and nicotinic acetylcholine receptor conotoxins [5]. As reported, most conotoxins are toxic to animals and humans, and the venom of *Conus geographus* (L.) can actually cause human death (respiratory failure) by bites or stings [6].

The mature αB-CTX contains 40 amino acids, belonging to the B3 superfamily, with four cysteines in the framework, and there are two disulfide bonds, forming a specific disulfide format (C–CC–C) [7]. αB-CTX is a novel and important nicotinic acetylcholine receptor (nAChR) antagonist, with the greatest potency against the α9α10 subtype [7], and it is worth noting that the α9α10 nAChR is an important target for the development of analgesics and cancer chemotherapeutics [8,9,10,11]. Moreover, αB-CTX can be used as a novel ligand to probe the structure and function of the α9α10 nAChR. At present, the main analytic and identification methods of αB-CTX are based on analytic chemistry, including NMR spectroscopy [7] and circular dichroism analysis [7,12]. However, these methods are time consuming, costly, and need trained personnel and complex sample pre-treatment [13]. For these reasons, it is essential to develop a rapid, accurate and sensitive method for αB-CTX identification in drug and real samples. Moreover, a fast and reliable detection method, based on an antibody of αB-CTX in real samples, was not reported until now. The above condition strongly demonstrated that the development of an effective detection method for αB-CTX analysis is necessary and valuable. Recently, immunochromatographic strips with high specificity and practicability, based on a monoclonal antibody (mAb), have been used to rapidly detect various toxins in real samples [14]. Compared to the traditional instrument detection methods, detection using immunochromatographic strips can be finished in several minutes (5~10 min), without any equipment or trained personnel [13,15]. Colloidal gold is used as a signal reporter in immunochromatographic strips for the detection of toxins in real samples [16], since it can be observed by the naked eye and is easy to synthesize [13]. To improve the sensitivity of detection, gold nanoflower particles, with a larger surface area and higher signal intensity, were synthesized and used for detection [17,18,19]. In our previous study, we prepared the mAb 5E4 against αB-CTX, with high affinity and specificity [20], in our lab, and the purified mAb 5E4 was used to construct the AuNPs-/AuNFs-based strips for αB-CTX detection in real samples.

## 2. Results and Discussion

### 2.1. Titer Determination of Anti-αB-CTX IgG2b Antibody

**Figure 1:**
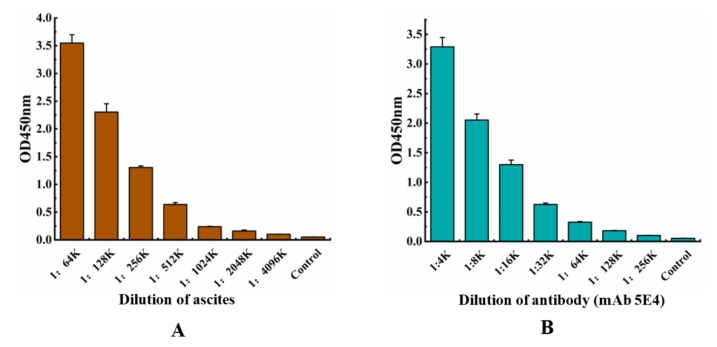
Titer determination of anti-αB-CTX mAb 5E4. (**A**) The titer of ascites. (**B**) The titer of purified anti-αB-CTX mAb 5E4.

The ascites was collected from the mouse after injection of the cultured hybridoma 5E4 cells, and the anti-αB-CTX mAb 5E4 (IgG2b) was further purified from the ascites by affinity chromatography, using a protein G column. The purified mAb 5E4 was analyzed by SDS-PAGE, and the result showed that there were two obvious protein bands at 50 kDa and 25 kDa, corresponding to the heavy chain and light chain, respectively, further indicating that the purification of mAb 5E4 was successful (data not shown). The concentration of the purified mAb 5E4 was 1.33 mg/mL. To determine the titer of the purified antibodies, indirect ELISA (iELISA) was performed, with the TRX-αB-CTX as a coating antigen. As shown in Figure 1A,B, the titer of the purified antibody was about 16,000, and it has similar antigen-binding activity to ascites, demonstrating that the anti-αB-CTX mAb 5E4 has high binding activity to αB-CTX.

### 2.2. Construction and Identification of AuNPs-Based Strip

**Figure 2:**
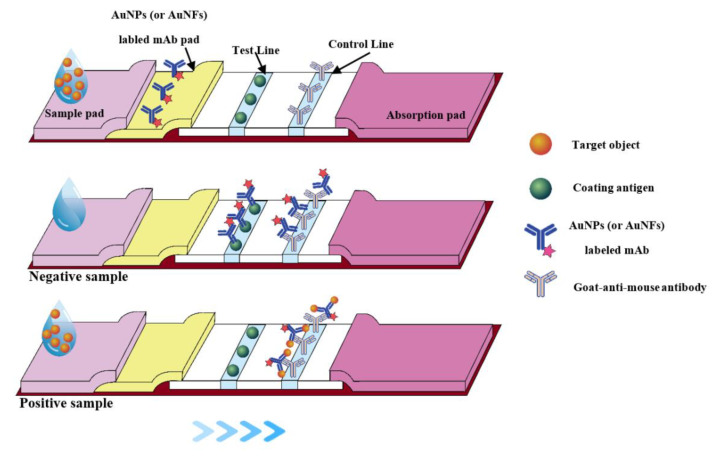
Schematic diagram of the AuNPs-based strip (or AuNFs-based strip).

**Figure 3:**
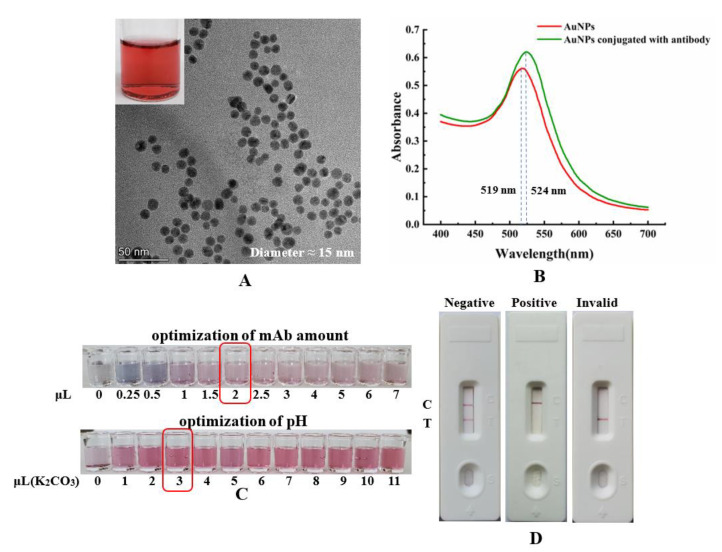
Characterization of AuNPs solution and construction of AuNPs-based strips. (**A**) Transmission electron microscopy (TEM) image of the AuNPs. (**B**) UV–visible absorption spectra scan of AuNPs and AuNPs conjugated with mAb. (**C**) Optimization of reaction solution parameters. The top: optimization of mAb amount, the volumes of mAb solution were 0, 0.25, 0.5, 1, 1.5, 2, 2.5, 3, 4, 5, 6 and 7 μL, respectively (left to right). The bottom: optimization of solution pH, added volumes of 0.1 M K_2_CO_3_ solution were 0, 1, 2, 3, 4, 5, 6, 7, 8, 9, 10 and 11 μL, respectively (left to right). (**D**) The effectiveness of the prepared AuNPs-based strip, C: control line; T: test line.

Colloidal gold nanoparticles (AuNPs) could easily be observed to conjugate with the antibody. The immunochromatographic strip test, based on colloidal gold nanoparticles, was successfully applied in the detection [21]. A schematic diagram of the immunochromatographic strip test is shown in Figure 2, to explain the working mechanism. To establish the AuNPs-based strip for αB-CTX detection, colloidal gold nanomaterials were prepared successfully (Figure 3A, transmission electron microscopy (TEM) image of the AuNPs). The color of the solution changed to a stable wine-red color, and the result derived from the TEM image showed that the average diameter of these particles was approximately 15 nm, and that they were well dispersed (Figure 3A). As shown in Figure 3B, the prepared colloidal gold solution had a maximum absorption wavelength of 519 nm, and the high-quality product could be used for further study. In the process of conjugation, the amount of labeled antibody and the pH of the reaction solution are important parameters that affect the accuracy of further study required. The result in Figure 3C showed that the optimal amount of anti-αB-CTX mAb was 2 μL mAb per 200 μL colloidal gold solution, in order for the reaction to be observed by the naked eye. For pH optimization, 0.1 M potassium carbonate (K_2_CO_3_) was used to adjust the pH of the solution. The optimal pH for mAb 5E4 and the colloidal gold conjugation, without precipitation, was adjusted by adding 3 μL 0.1 M K_2_CO_3_ into 200 μL solution, under naked eye observation. Creating the AuNPs–antibody conjugation is the key step, and the absorbance peak of the resulting conjugates was analyzed by UV (Figure 3B). As expected, the maximum absorbance peak of the solution changed from 519 nm to 524 nm, further indicating that the AuNPs–antibody conjugation was prepared successfully. Then, the AuNPs-based strip was constructed by using the AuNPs–antibody conjugation, and a schematic diagram of the immunochromatographic strip test is shown in Figure 2, to explain the working mechanism. As shown in Figure 3D, if there was little or no toxin to react with the AuNPs–mAb 5E4 conjugate, two red bands appeared on the nitrocellulose (NC) membrane, indicating the negative result. In the positive tests, there was a sufficient amount of toxin to react with the AuNPs-labeled mAb 5E4, and the conjugate could not be captured by the coating antigen, failing to form the test line. Therefore, the result showed that only one red band appeared on the control line. If the red color only appeared on the test line, without a visible red color present on the control line, the test was considered to be invalid. The above results also clearly showed that the AuNPs–antibody probe was successfully conjugated and could be used in further study.

### 2.3. Specificity and Sensitivity Analysis of AuNPs-Based Strip

**Figure 4:**
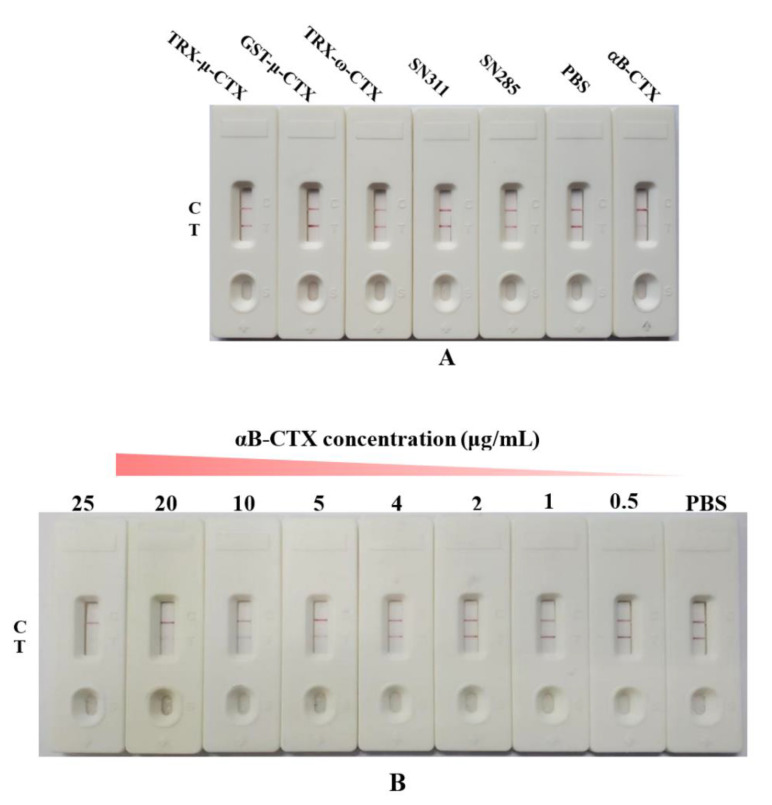

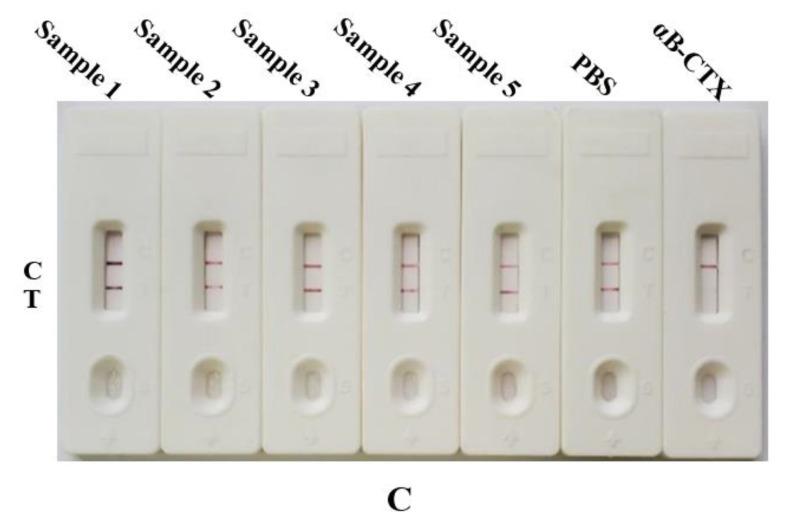
Characterization of AuNPs-based strips and detection of αB-CTX in real samples. (**A**) Specificity of AuNPs-based strip with other correlated antigens. (**B**) The sensitivity and visual detection limit (vLOD) of AuNPs-based strip by the naked eye. (**C**) The actual samples were detected by AuNPs-based strip. Samples 1 to 5 are shellfish (qīng é), *Viviparidae*, *Thais clavigera Kuster*, *Oncomelania hupensis Gredler* and *Ruditapes philippinarum*, respectively.

The specificity of the AuNPs-based strip was evaluated by adding other toxins into different sample pads, such as the fusion proteins thioredoxin-µ-conotoxin KIIIA (TRX-μ-CTX) and glutathione S-transferase-µ-conotoxin KIIIA (GST-μ-CTX), as well as thioredoxin-ω-conotoxin MVIIA (TRX-ω-CTX), sea snake venom 311 (SN311) and sea snake venom 285 (SN285). μ-CTX and ω-CTX are two different conotoxins secreted by cone snails, and they are closely related to αB-CTX. TRX-μ-CTX and GST-μ-CTX indicate that μ-CTX has fused with TRX and GST tags, respectively, and TRX-ω-CTX is the fusion protein of TRX and ω-CTX. Except for conotoxins, other related toxins, such as sea snake SN311 and SN285, were used to test the specificity of the developed AuNPs-based strip. Compared to the other correlated toxins, only one red band appeared on the control line in the strip of αB-CTX (Figure 4A), showing that the strip test had high specificity to αB-CTX. Meanwhile, the sensitivity of the strip was further analyzed by dropping αB-CTX into sample pads with different concentration ranges, from 0.5 to 25 μg/mL. According to the published paper [13,22], vLOD was defined as the lowest concentration of target solution that causes the color of the test line on the NC membrane to become weaker, when compared to the negative control (sample solution without any target). In Figure 4B, the vLOD of the AuNPs-based strip for αB-CTX detection was determined to be 4 μg/mL, and the concentration of dropped αB-CTX solution was 25 μg/mL when the test line disappeared completely. The result indicated that αB-CTX (25 μg/mL) was sufficient to completely bind the mAb–AuNPs conjugates on the pad. The time of visual evaluation was less than 10 min.

### 2.4. AuNPs-Based Strip for αB-CTX Detection in Real Samples

We aimed to further study the accuracy and practicability of the established AuNPs-based strip. Five different kinds of actual snail or shellfish samples (*Oncomelania hupensis Gredler*, shellfish (qīng é), *Ruditapes philippinarum*, *Viviparidae* and *Thais clavigera Kuster*) were purchased randomly from the Fuzhou markets in China. These samples were pre-treated by smashing them with PBS solution before filtering, and the αB-CTX was obtained in soluble form by collecting the supernatant. The results in Figure 4C showed that no αB-CTX was detected in these samples (similar to the negative control).

### 2.5. Construction and Identification of AuNFs-Based Strip

**Figure 5:**
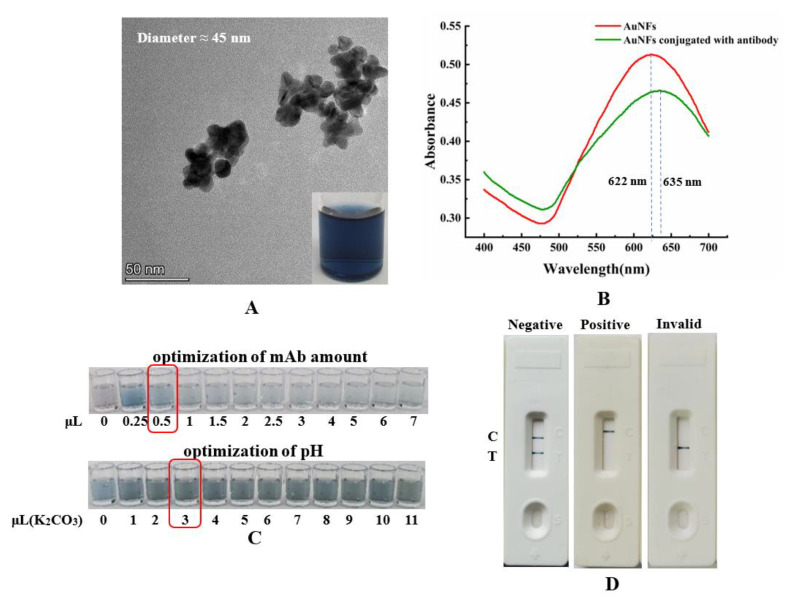
Preparation of AuNFs solution and construction of AuNFs-based strip. (**A**) Transmission electron microscopy (TEM) image of the AuNFs. (**B**) UV–visible absorption spectra scan of AuNFs and AuNFs conjugated with mAb. (**C**) Optimization of reaction solution parameters for AuNFs. The top: optimization of mAb amount, the volumes of mAb solution were 0, 0.25, 0.5, 1, 1.5, 2, 2.5, 3, 4, 5, 6 and 7 μL, respectively (left to right). The bottom: pH optimization of solution, the volumes of 0.1 M K_2_CO_3_ solution were 0, 1, 2, 3, 4, 5, 6, 7, 8, 9, 10 and 11 μL, respectively (left to right). (**D**) The effectiveness of prepared AuNFs-based test strips, C: control line; T: test line.

The successfully prepared AuNPs, with an average diameter of 15 nm, were used as the signal reporters of the AuNPs-based strip, for rapidly detecting αB-CTX. It is well known that the size of the AuNPs is an important factor to improve the sensitivity of detection [23]. Nanoflower gold particles (AuNFs), with a multi-branched surface and large size, have been widely used as signal reporters to enhance the sensitivity of detection [17]. The AuNFs were prepared by using the seeding growth method, and the pH of the solution significantly affected the color of the solution and the shape of the AuNFs. A pH of 7.0 in the reaction solution was suitable for the synthesis of AuNFs, according to previous reports [17,23]. As shown in Figure 5A, the color of the nanoflower solution was deep blue, with good transparency and stability, and the prepared AuNFs showed a complex three-dimensional and flower-like shape under TEM observation. Meanwhile, the average diameter of the AuNFs was measured to be about 45 nm, which is within the range of nanoparticles suitable for labeling antibodies [17]. As shown in Figure 5B, the prepared nanoflower solution, with a maximum absorption wavelength of 622 nm, confirmed that the AuNFs were successfully prepared by using the seeding growth method. Similar to the AuNPs, optimization of the amount of labeled antibody and the pH of the reaction solution is necessary for AuNFs-based strip construction. First, the optimal amount of mAb was determined to be 0.5 μL mAb per 200 μL nanoflower solution, in order for the reaction to be observed by the naked eye (Figure 5C). However, the result in Figure 5C showed that adding 3 μL 0.1 M K_2_CO_3_ into 200 μL nanoflower solution was optimal to adjust the pH for the AuNFs–mAb conjugation, without precipitation. The UV–visible absorbance result is shown in Figure 5B; the prepared AuNFs–mAb 5E4 conjugates had a maximum peak at 635 nm, and created an obvious shift compared to that of the AuNFs solution, further indicating that the mAb 5E4 was successfully labeled with the AuNFs. The detection principle of the AuNFs-based strip was similar to that of the AuNPs-based strip. If two blue bands appeared on the nitrocellulose (NC) membrane, it could be reasoned that no toxin in the sample had reacted with the AuNFs-labeled antibody, indicating a negative result (Figure 5D). If the content of αB-CTX was sufficient to react with the AuNFs-labeled antibody, only one blue band appeared on the control line, indicating that the sample is positive. When the blue band only appeared on the test line, without a visible band present on the control line, the test was considered to be invalid (Figure 5D). The synthesis of AuNFs-based test strips with mAb is usually carried out in a dry (humidity <25%) and warm environment (25 °C, room temperature), and the total process of the synthesis of AuNFs-based test strips can be easily finished in one day under normal conditions. The above results also clearly showed that the AuNFs-based strip was assembled successfully, and that it can be used for further study.

### 2.6. Specificity and Sensitivity Analysis of AuNFs-Based Strip

**Figure 6:**
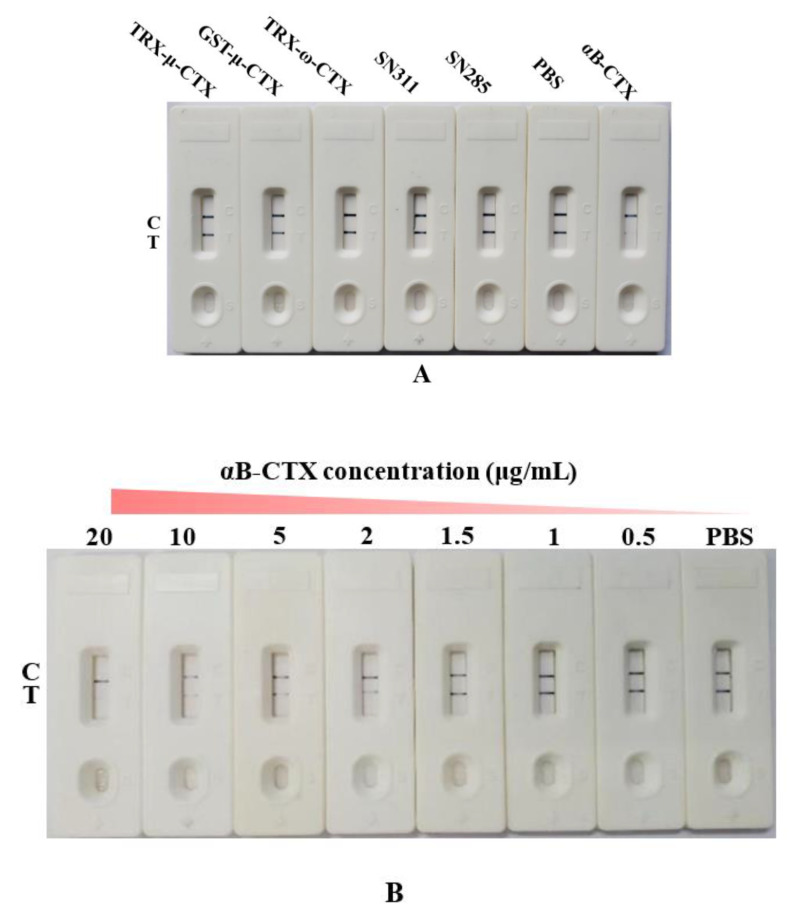

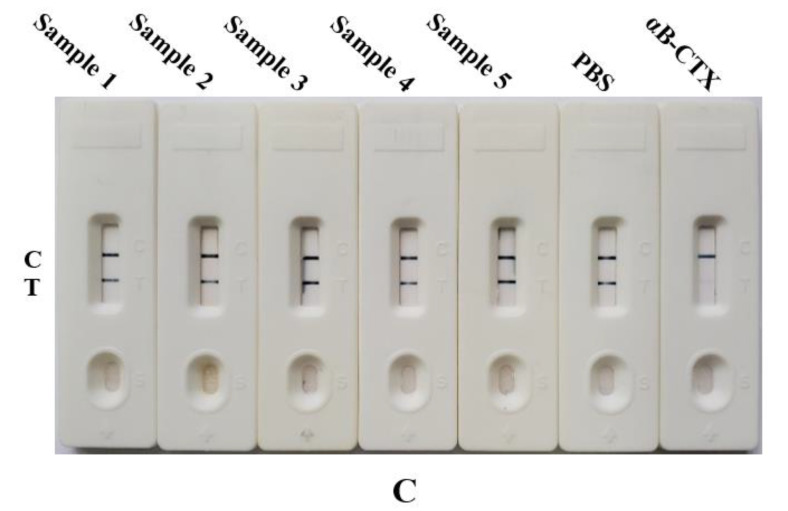
Characterization of AuNFs-based test strips and αB-CTX detection in real samples. (**A**) Specificity analysis of AuNFs-based strip with other correlated antigens. (**B**) The sensitivity and visual detection limit (vLOD) of AuNFs-based strip by the naked eye. (**C**) The actual samples were detected by AuNFs-based test strips. Samples 1 to 5 are shellfish (qīng é), *Viviparidae*, *Thais clavigera Kuster*, *Oncomelania hupensis Gredler* and *Ruditapes philippinarum,* successively.

To evaluate the specificity of the AuNFs-based strip, some correlated toxins (TRX-μ-CTX, GST-μ-CTX, TRX-ω-CTX, SN311 and SN285) were used as the antigen to compete the AuNFs-labeled antibody with TRX-αB-CTX, and the detection result is shown in Figure 6A. The prepared AuNFs-based strip exhibited excellent specificity, and two blue bands appeared in all the strips of the correlated toxins, except for that of αB-CTX (Figure 6B). In addition, the sensitivity of the constructed strip was analyzed by using different concentrations of αB-CTX (from 0.5 to 20 μg/mL). The vLOD of the AuNFs-based strip was 1.5 μg/mL, which was a smaller vLOD value than that of the AuNPs-based strip (4 μg/mL). This difference in vLOD can be easily explained by the fact that the AuNFs with a larger size and multi-branched surface resulted in more antibody coupling, further improving the sensitivity of target detection. Therefore, it is worth noting that the AuNFs-based strip for αB-CTX detection was successfully established in this study.

### 2.7. AuNFs-Based Strip for αB-CTX Detection in Real Samples

To further determine the accuracy and feasibility of the AuNFs-based strip developed in this study, five different kinds of actual snail or shellfish were purchased randomly from the Fuzhou markets in China. The protocols of sample pre-treatment and detection for the AuNFs-based strips were the same as those for the AuNPs-based strip. The results in Figure 6C showed that αB-CTX was not detected in these real samples, and the positive and negative controls all provided the correct results.

## 3. Conclusions

In the present study, the lateral flow immunoassays used for the detection and analysis of αB-CTX were constructed using the mAb 5E4, with high specificity. The vLOD values of the AuNPs-based strip and AuNFs-based strip were 4 and 1.5 μg/mL, respectively, with the whole process taking less than 10 min. To the best of our knowledge, this is the first report describing the development of immunosensors based on an antibody, to determine and analyze the content of αB-CTX in real samples. In view of the excellent signal stability, repeatability and accuracy of the detection methods, the AuNPs-/AuNFs-based strips developed in this study could be used for the identification and rapid detection of αB-CTX in real samples.

## 4. Materials and Methods

### 4.1. Materials

Standard αB-CTX (5 mg/mL, purity ≥98% by HPLC) was synthesized by Sangon Biotech (Shanghai, China). Fusion proteins, TRX-αB-CTX, TRX-μ-CTX, GST-μ-CTX and TRX-ω-CTX, were previously prepared in our lab. BCA protein assay kit was purchased from Solarbio Life Science (Beijing, China). Horseradish peroxidase (HRP)-labeled goat anti-mouse IgG antibody was purchased from Genscript Biotech Corporation (Nanjing, China). Hydroquinone, chloroauric acid (HAuCl_4_) and trisodium citrate were purchased from Shanghai Chemical Reagents (Shanghai, China). Nitrocellulose (NC) membrane, conjugate pad, absorbent pad and sample pad were purchased from Millipore Corporation (Bedford, MA, USA). All other chemicals were analytical grade, and were purchased from Beijing Chemical Reagent Co. (Beijing, China).

### 4.2. Preparation of Anti-αB-CTX mAb 5E4

Hybridoma cell line 5E4, which stably secreted anti-αB-CTX mAb, was previously prepared in our laboratory [20]. The cultured hybridoma cells (about 1.5 × 10^6^ cells) were injected into the abdomen of a pre-sensitized Balb/c mouse for the formation of ascites fluid. The mAb 5E4 with the subtype IgG2b was purified by protein G affinity chromatography, and the fineness of the purified antibody was analyzed by SDS-PAGE. BCA protein assay kit was used to determine the concentration of proteins [24], and the binding activities (titer) of ascites fluid and anti-αB-CTX mAb 5E4 were determined by iELISA [25].

### 4.3. Preparation of AuNPs

To achieve fast and accurate detection of αB-CTX in real samples, colloidal gold strip was prepared. The gold nanospheres (AuNPs) were prepared using the citrate reduction method, as described, with minor modifications [26,27]. Briefly, 100 mL of 0.01% chloroauric acid solution (HAuCl_4_) in distilled water was slowly heated to boiling point, and then 2.0 mL of 1% trisodium citrate solution was slowly added into the solution. The color of the solution changed from yellow to wine red; it was boiled continuously for 7 min, then allowed to gradually cool to room temperature. UV–visible absorptio spectrum was performed to analyze the absorbance peak of the solution at 400–700 nm. The particle shape was observed by a transmission electron microscope (TEM), and the average particle size was measured [28].

### 4.4. Preparation and Optimization of AuNPs–Antibody Conjugates

AuNPs–antibody conjugation was performed as described, with minor modifications [23]. The prepared colloidal gold solution (about 200 μL) was added into the series of 96-well plates separately, and different volumes of purified mAb solution (from 0 to 7 μL) were added into the colloidal gold solution. After incubating for 30 min, 20 μL of 10% NaCl solution was added into the mixture, and the minimum volume of mAb that maintained the red color in the well without change was considered the optimal volume of mAb. The pH of colloidal gold solution was adjusted with potassium carbonate (0.1 M K_2_CO_3_), and the steps were similar to those described above. The optimal pH was defined as the corresponding volume of K_2_CO_3_ when the color of the solution was maintained red, without change.

### 4.5. Construction of AuNPs-Based Strips

After optimization of pH and antibody volume, the colloidal gold–antibody conjugate sediment collected by centrifugation was resuspended in dilution buffer (0.01 M phosphate buffer, 1% BSA (*w/v*), 0.5% PEG-20000 (*w*/*v*)) and stored at 4 °C for further use. This test strip consisted of four pads, including sample pad, AuNPs-labeled antibody pad, nitrocellulose membrane (NC) and absorption pad [28]. TRX-αB-CTX was the coating antigen and was fixed to the NC membrane to form the test line, and goat anti-mouse IgG was immobilized on the T line to capture the AuNPs-labeled antibody in the process of liquid migration. To assemble the AuNPs-based strip, the sample pad and AuNPs-labeled antibody pad were soaked in blocking buffer (0.01 M PBS, 5% BSA (*w*/*v*), 1% Tween-20 (*v*/*v*)) for 2 h and then incubated at 37 °C overnight. Then, the assembled strip complex was cut into 5 mm-wide strips using programmable Sheet Cutter CTS300 (Kinbio Tech. Co., Ltd., Shanghai, China) for further use.

### 4.6. Specificity and Sensitivity Analysis of AuNPs-Based Strip

To determine the specificity of the test strip, five antigens, correlated to αB-CTX, such as fusion protein TRX-μ-CTX, GST-μ-CTX, TRX-ω-CTX, SN311 and SN285, were added into the sample pad of the test strip, respectively, and all antigens were adjusted to the same concentration (25 μg/mL). To assess the sensitivity of the test strips, different concentrations of αB-CTX antigen (0.5, 1, 2, 4, 5, 10, 20 and 25 μg/mL) were allowed to react with the AuNPs-labeled antibody, and the sensitivity and limit of detection of the αB-CTX antigen can be observed according to the T and C line results from the colloidal gold strips [29].

### 4.7. Preparation and Optimization of AuNFs–Antibody Conjugation

To enhance the sensitivity of αB-CTX detection, nanoflower gold particles (AuNFs) were prepared by using the gold seeding method, with slight modifications [27,30]. The mixture of sodium citrate, hydroquinone and HAuCl_4_ solution was used to prepare the AuNFs, with the AuNPs as gold seeds (100 μL), under reduced conditions. HAuCl_4_ solution (0.01%, 750 μL) and 300 μL 1% trisodium citrate solution were added into 100 mL deionized water with constant stirring. Then, the pH of the reaction solution was adjusted to 7.0, and hydroquinone (0.03 mol/L, 750 μL) was added into the reaction solution drop by drop until the color of the solution changed to dark blue, then the solution was stored at 4 °C for subsequent use. The absorbance value of the solution at 400–700 nm was determined using an ultraviolet spectrophotometer, and the shape and size of AuNFs were observed and measured using an transmission electron microscope (TEM). To assemble the AuNFs-based strip, the pH and antibody volume were optimized, and all the steps of optimization were the same as those for the AuNPs-based strip, as described in the above section [27].

### 4.8. Construction and Identification of AuNFs-Based Strip

To construct the AuNFs-based strip, the AuNFs probe was prepared according to the reported studies [23], and all the steps of preparation were similar to those of the AuNPs–antibody conjugates described in the above section. The specificity and sensitivity of the AuNFs-based strip were characterized using the same protocols as the colloid gold strip.

### 4.9. αB-CTX Detection by AuNPs-/AuNFs-Based Strip in Real Samples

To evaluate the accuracy of the strip test, five different kinds of actual snail or shellfish samples were purchased randomly from the Fuzhou markets in China. The collected samples were mashed and centrifuged at 10,000 r/min for 5 min [31], and the supernatant was used for evaluating the αB-CTX residue in real samples by the developed AuNPs-/AuNFs-based strip. All the steps of detection were the same as described above.

## Data Availability

Not applicable.
